# Pegylated Interferon and Ribavirin Treatment for Hepatitis C Virus Infection

**DOI:** 10.1155/2010/275274

**Published:** 2011-01-04

**Authors:** Tatehiro Kagawa, Emmet B. Keeffe, Ming-Lung Yu

**Affiliations:** ^1^Department of Gastroenterology, Tokai University School of Medicine, 143 Shimokasuya, Isehara 259-1193, Japan; ^2^Division of Gastroenterology and Hepatology, Department of Medicine, Stanford University Medical Center, Palo Alto, CA 94304-1509, USA; ^3^Department of Internal Medicine, Kaohsiung Municipal Ta-Tung Hospital, Kaohsiung Medical University Hospital, Kaohsiung Medical University, Kaohsiung 801, Taiwan

We are pleased to serve as editors of this special issue. We were gratified by the excellent response to the call for papers and the high quality of the eleven manuscripts that were ultimately accepted for this special issue.

This issue begins, as is appropriate, with a historical perspective by R. M. Friedman and S. Contente from the Uniformed Services University of the Health Sciences on treatment of chronic hepatitis C with interferon, followed by a paper by C.-H. Chen and M.-L. Yu from Kaohsiung Medical University tracing the evolution of interferon-based therapy, including the addition of ribavirin and pegylation of interferon. Both of these papers provide an interesting capsule of where we began with the initial recognition of the antiviral activity of interferon in 1957, followed by the evolution interferon-based therapy of chronic hepatitis C with the addition of ribavirin in the mid-1990s and the demonstration of the superiority of pegylated interferon (peginterferon) plus ribavirin in the early 2000s. The sustained virologic response (SVR) rate increased from 8%–9% with interferon monotherapy to 30% in genotype 1 patients with the addition of ribavirin, and then to 40%–50% with the transition to peginterferon plus ribavirin. As noted in several papers in this special issue, we are on the threshold of increasing the SVR rate in 2011 up to 65%–75% with the addition of a protease inhibitor to the standard of care with peginterferon plus ribavirin. Thus, advances in the treatment of chronic hepatitis C continue to march forward, with SVR rates increasing from less than 10% with interferon monotherapy to close to 75% in the very near future with triple therapy using a protease inhibitor. What is remarkable is that this progress has all taken place within the past 20 years. In the next paper, T. Kagawa and E. Keeffe review the recent literature evaluating the long-term outcomes of antiviral therapy in patients with chronic hepatitis C that convincingly demonstrate the impact of interferon-based treatment of chronic hepatitis C. The published data shows slowed disease progression in patients who achieve an SVR, including improved inflammation and fibrosis scores on follow-up biopsy, reduced incidence of hepatocellular carcinoma, and prolonged life expectancy with reduced liver-related deaths. Thus, the progress in treatment over the past 20 years is paying dividends to our patients.

The prediction of response to interferon-based therapy is an important issue for patients considering embarking on a course of therapy, and providers of care continue to improve their ability to predict success in individual patients by taking into account baseline host (age, race, gender, histology (stage of fibrosis and presence or absence of steatosis), body weight, insulin resistance, and *IL28B* genotype) and viral factors (genotype and HCV RNA level). A number of papers in this special issue address predictors of viral response. N. Izumi et al. provide a thorough and scholarly review of the various factors that predict an SVR and their relative importance. R. Cubillas et al. from Southwestern Medical Center in Dallas elegantly demonstrate that tumor necrosis factor receptor 1 is upregulated in dendritic cells in patients with chronic hepatitis C who respond to therapy. Although not clinically available, R. Kubota et al. demonstrate that erythrocyte ribavirin levels can predict which patients are more likely to achieve an early virologic response after 12 weeks of treatment. 

A number of miscellaneous papers in this special issue address the treatment of chronic hepatitis C in a number of special populations. M. Numata et al. from several Japanese medical centers treated 122 patients with genotype 1 infection and demonstrated by multivariate analysis that adherence to peginterferon and ribavirin was the only predictor of SVR. The rate of SVR fell sharply when exposure to peginterferon was less than 80% and also decreased in a stepwise fashion when ribavirin exposure was 60%–80% and less than 60% compared with greater than 80%. D. F. Meyer et al. from several New York medical centers report that daily high-dose consensus interferon (24 *μ*g) plus weight-based ribavirin was not successful in genotype 1 nonresponders to prior therapy and was associated with substantial side effects. Y. Y. Hwang and R. H. S. Liang from Queen Mary Hospital in Hong Kong review antiviral therapy in hematologic patients and point out that the published literature demonstrates that HCV RNA levels increase during chemotherapy and immunosuppression and that there is a risk of rebound immunity against hepatitis C with liver injury after discontinuation of immunosuppression. They recommend that close monitoring during chemotherapy is appropriate and that antiviral therapy with peginterferon and ribavirin should be deferred until complete of chemotherapy and recovery of immunity. H. Weclawiak et al. focus on the management of chronic hepatitis C during hemodialysis and reinforce the recommendation to treat during dialysis, as there is a high rate of SVR that eliminates recurrence of HCV infection after kidney transplantation. They also reinforce the standard recommendation not to treat chronic hepatitis C after kidney transplantation because of the high risk of acute allograft rejection. Finally, Y. Sugawara et al. from the University of Tokyo provide a thorough review of the controversies and challenges in the management of recurrent chronic hepatitis C after liver transplantation. Although there is no general consensus on who, when and how to treat, the overall SVR rate is 25%–45% with standard peginterferon plus ribavirin, in spite of a high prevalence of intolerability. The newer antiviral therapies in 2011 may bring greater success in the management of patients with posttransplant recurrent HCV infection.

The editors are confident that the readers will enjoy reading this special issue and the diverse topics that are expertly reviewed. 

*Tatehiro Kagawa* *Tatehiro Kagawa* *Emmet B. Keeffe* *Emmet B. Keeffe* *Ming-Lung Yu* *Ming-Lung Yu*

